# Supplementary material for: Illumina SBS Sequencing and DNBSEQ Perform Similarly for Single-Cell Transcriptomics
Source: Genes (Basel). 2024 Nov 6;15(11):1436. doi: 10.3390/genes15111436 (PMC11594097; doi:10.3390/genes15111436)
Supplement: Supplementary file 1 [file genes-15-01436-s001.zip › genes-3244835 - supplemental figure 1 with legend.pdf]

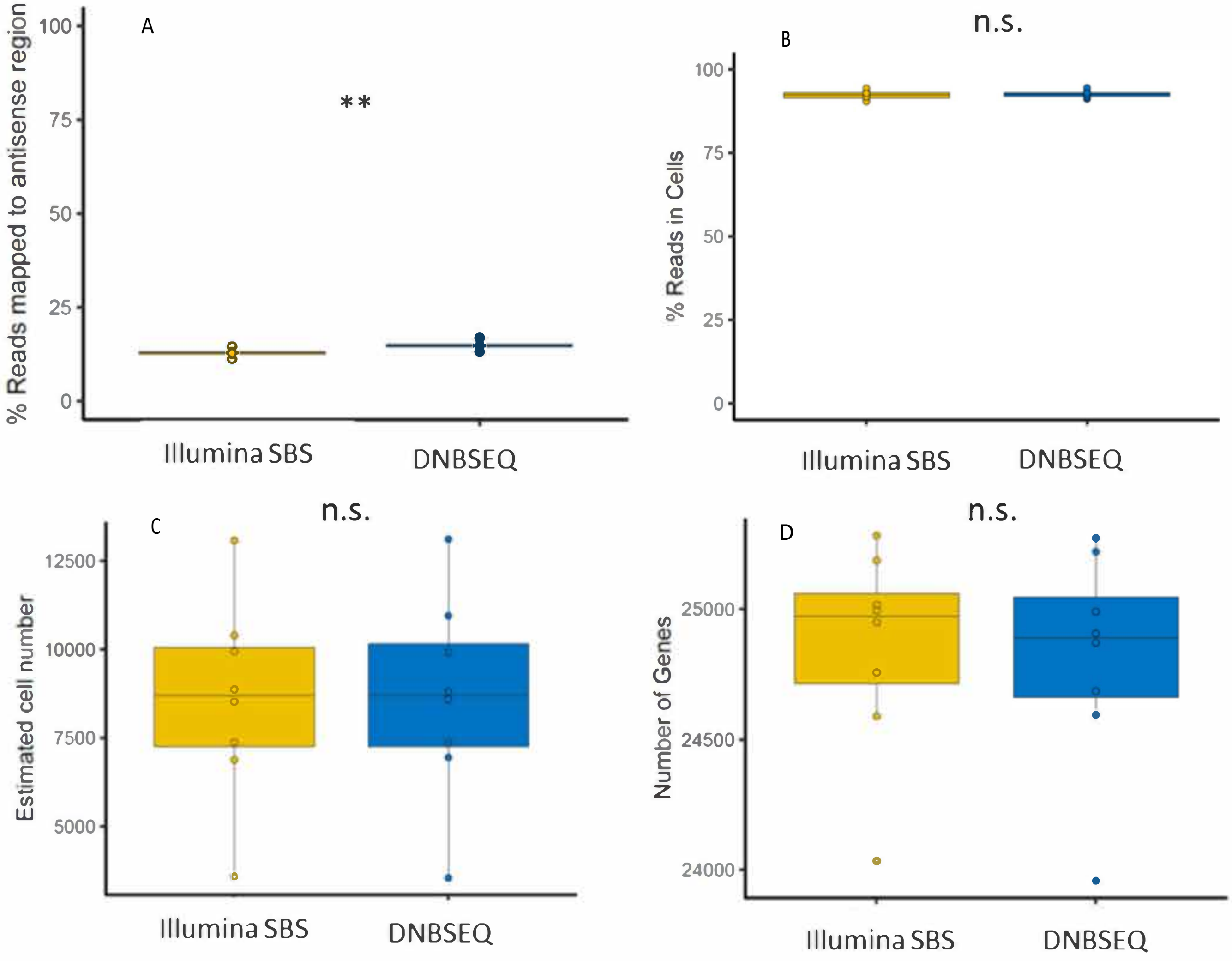


**Supplemental 1 DNBSEQ has more reads mapped to the antisense regions than Illumina SBS. The single-cell related metrics - i.e. the percentage of reads in cells, the number of cells and the number of genes - were not altered depending on the technology used.**

A) Box plot with the percentage of reads confidently mapped to the antisense strand genome regions (n = 8; Illumina SBS mean ± SD = 12.8 ± 0.9, DNBSEQ mean ± SD = 14.8 ± 1.1; t-test p-value= 0.00159) B) Boxplot representing the percentage of reads in cells (n = 8; Illumina SBS mean ± SD = 92.5 ± 1.1, DNBSEQ mean ± SD = 92.2 ± 1.2; p-value=0.632) C) Boxplot representing the estimated number of cells retrieved per sample (n=8; Illumina SBS mean ± SD = 8578 ± 2796, DNBSEQ mean ± SD = 92.2 ± 1.2; p-value=0.961) D ) Boxplot with the average number of genes detected per sample (n = 8; Illumina SBS mean ± SD = 24852 ± 397, DNBSEQ mean ± SD = 24812 ± 417; p-value = 0.851).
